# Efficacy of Two Antibiotic-Extender Combinations on *Mycoplasma bovis* in Bovine Semen Production

**DOI:** 10.3390/pathogens9100808

**Published:** 2020-09-30

**Authors:** Tarja Pohjanvirta, Nella Vähänikkilä, Henri Simonen, Sinikka Pelkonen, Tiina Autio

**Affiliations:** 1 Finnish Food Authority, Laboratory and Research Division, Veterinary Bacteriology and Pathology Unit, Neulaniementie 4, 70210 Kuopio, Finland; nella.vahanikkila@foodauthority.fi (N.V.); sinikka.pelkonen@foodauthority.fi (S.P.); tiina.autio@foodauthority.fi (T.A.); 2 VikingGenetics, Korpikyläntie 77, 15870 Hollola, Finland; hesim@vikinggenetics.com

**Keywords:** *Mycoplasma bovis*, bovine semen, antibiotics, prevention, DNA extraction

## Abstract

*Mycoplasma bovis* is an important bovine pathogen. Artificial insemination (AI) using contaminated semen can introduce the agent into a naïve herd. Antibiotics, most often gentamycin, tylosin, lincomycin, spectinomycin (GTLS) combination are added to semen extender to prevent transmission of pathogenic bacteria and mycoplasmas. In a commercial AI straw production system with industrial scale procedures, we analyzed the mycoplasmacidal efficacy of GTLS and ofloxacin on *M. bovis* ATCC and wild type strain isolated from commercial AI straws. The strains were spiked at two concentrations (10^6^ and 10^3^ CFU/mL) into semen. Viable *M. bovis* in frozen semen straws was detected by enrichment culture and real-time PCR. We also compared different protocols to extract *M. bovis* DNA from spiked semen. None of the antibiotic protocols had any effect on the viability of either of the *M. bovis* strains at high spiking concentration. At low concentration, the wild type was inhibited by all other protocols, except low GTLS, whereas the ATCC strain was inhibited only by high GTLS. The InstaGene™ matrix was the most effective method to extract *M. bovis* DNA from semen. When there is a low *M. bovis* contamination level in semen, GTLS used at high concentrations, in accordance with Certified Semen Services requirements, is more efficient than GTLS used at concentrations stated in the OIE Terrestrial Code.

## 1. Introduction

*Mycoplasma bovis* is a major bovine pathogen causing substantial economic losses and has a debilitating effect on animal welfare. *M. bovis* causes a variety of diseases including mastitis, pneumonia, arthritis, otitis media, and genital infections [1]. Efforts to develop efficacious vaccines have not been successful [2]. Once established in a cattle farm, *M. bovis* can be difficult to eradicate [3]. Consequently, it is of paramount importance to prevent the introduction of the agent into naïve herds.

One *M. bovis* transmission route into a herd is artificial insemination (AI) [4]. Recently, we reported on how contaminated semen used in AI, introduced *M. bovis* infection into closed naïve dairy herds [5]. In a previous study, heifers inseminated with semen containing *M. bovis* became repeat breeders, and only half of them finally conceived [6]. *M. bovis* could be isolated from cervico-vaginal mucus of some of the heifers, 8–32 weeks after insemination. Kissi et al. [7] showed that insemination with frozen *Mycoplasma sp*. containing semen often resulted in prolonged diestrus, suggesting that mycoplasma could initiate a pathological process in the uterus. However, very little is known about the concentration of *M. bovis* in naturally infected bull semen and the infectious dose needed to initiate an infection in the female genital system. 

There are several viral and bacterial pathogens that can be transmitted via semen [8]. Semen used for AI should be free of infectious agents. Several types of antibiotics have been added to seminal extenders before freezing to control bacterial contamination, including mycoplasmas. The World Organization for Animal Health OIE lists, in the OIE Terrestrial Code [9], the following three different antibiotic combinations to be used in international trade of bovine semen: gentamicin (250 µg), tylosin (50 µg), lincomycin-spectinomycin (150/300 µg) (GTLS) in each mL of frozen semen; penicillin (500 IU), streptomycin (500 µg), lincomycin-spectinomycin (150/300 µg) (PSLS); or amikacin (75 µg), divekacin (25 µg). The European Union directive 88/407/1993 includes the use of the above mentioned PSLS, or an alternative combination of antibiotics with an equivalent effect against campylobacters, leptospires, and mycoplasmas. Shin et al. [10], in 1988, developed a method where GTLS concentration was doubled as compared with the concentration stated in OIE Code, and GTLS was first added into raw semen before extending with GTLS containing extender. Nowadays, GTLS is widely used in bovine semen production, and Certified Semen Services (CSS) in USA has a special protocol in place for GTLS use [11]. However, Visser et al. conducted two studies, in 1995 and 1999 [12,13], in which they questioned the ability of even the high GTLS concentration to control *M. bovis* in AI. Since the studies of Shin et al. [10] and Visser et al. [12,13], animal protein sources in commercial extenders have often been replaced with plant protein sources such as soybean lecithin to avoid disease transmission through the use of animal source protein [14]. Most commercially available soy-lecithin-based extenders contain GTLS as standard antibiotics. However, recent *M. bovis* isolates have shown a marked increase in MIC90 values for tylosin, lincomycin, and spectinomycin, but resistance against fluoroquinolones is still quite rare [15,16,17]. Recently a fluoroquinolone antibiotic, ofloxacin, was shown to be non-toxic to spermatozoa and effective in protecting semen from bacteria, although the authors did not analyze its effect on mycoplasmas [18]. 

Introductions of *M. bovis* into countries free of the organism have recently been reported (Finland 2012 [19], New Zealand 2017, (https://www.mpi.govt.nz/protection-and-response/mycoplasma-bovis/). Although these introductions have not been directly linked to semen, *M. bovis* risk, due to global semen trade, continues to be a concern, especially in New Zealand where eradication of *M. bovis* has been attempted. In this study, we evaluated the efficacy of the low OIE Code and the high CSS guideline GTLS concentrations and two ofloxacin concentrations on the viability of two different *M. bovis* strains in spiked frozen semen. We used an ATCC strain, as well as a wild type strain recently isolated from commercial AI semen straws [5]. Unlike in previous GTLS efficacy studies [10,12,13], we used a commercial animal protein free extender. We wanted to study if it was possible to achieve mycoplasmacidal effect, in other words, no detection of *M. bovis* in AI semen straws after semen was enriched in mycoplasma broth, and an aliquot of the broth culture was directly analyzed using *M. bovis* real-time polymerase chain reaction (PCR). 

Mycoplasmas are fastidious organisms needing special culture media and expertise in isolation. Instead of mycoplasma culture, PCR could be an option in AI centers to ensure *M. bovis*-free semen lots. There are only a few studies about PCR detection of *M. bovis* in bovine semen. Therefore, we also evaluated sensitivity of different DNA extraction methods to detect *M. bovis* in semen. 

Experiments to produce *M. bovis* contaminated AI straws were conducted, in a commercial AI straw producing laboratory, using industrial scale procedures. This was possible because semen production ceased in this center after these experiments.

## 2. Results

Raw pooled semen showed no growth in mycoplasma culture. *M. bovis* or Friis broth did not have any detrimental effect on quality parameters of semen (Table 1).

After storage of the AI straws for five weeks in liquid nitrogen, at high spiking concentrations (10^6^ CFU/mL), viable *M. bovis* bacteria were detected in processed semen regardless of the processing protocol. When low *M. bovis* concentrations were inoculated, differences among processing protocols were seen (Table 2). At a low spiking concentration, the ATCC strain was more resistant than the wild type strain to different antibiotics. The only protocol inhibiting the growth of the ATCC strain was the high GTLS 500/100/300/600 µg/mL (final concentration in extended semen) supplement added in the semen lab to the extender. All protocols, except EU GTLS 250/50/150/300 µg/mL (final concentration in extended semen) and extender without antibiotics, inhibited the growth of the wild type at a low spiking concentration. 

Antimicrobials present in extended semen affect the mycoplasma culture, and thus several dilutions were made. In samples with high concentration of antimicrobials, viable *M. bovis* could be detected only in the highest culture dilution (Table 2).

We compared three different DNA extraction methods for spiked semen samples. At a high spiking concentration (10^6^ CFU/mL), all pools were positive in PCR regardless of the DNA extraction method. Ct values varied between 24.7 and 28.5, and no significant differences in Ct values among extraction methods were seen (data not shown).

At a low spiking concentration, the method using InstaGene™ (method three) was the most effective. Using this method, we detected *M. bovis* in 94% (17/18) of pools spiked with 10^3^ CFU/mL of ATCC strain, and in 72% (13/18) spiked with 10^3^ CFU/mL of wild type strain. With method one, 67% (12/18) and with method two, 56% (10/18) of pools spiked with ATCC strain were positive in PCR, respectively. For the wild type strain, respective figures were for method one 61% (11/18) and 33% (6/18) for method two (Table 3). The Ct values varied between 34.3 and 36.7, and no significant differences in Ct values among extraction methods were seen (data not shown).

Minimum inhibitory concentration values of the strains used in spiking are shown in Table 4**.**

## 3. Discussion

Our study showed that it is challenging to rely on the use of antibiotics in bovine semen production to control *M. bovis.* None of the studied antibiotics had any effect on viability of *M. bovis* at 10^6^ CFU/mL in extended semen, and the lower spiking concentration of 10^3^ CFU/mL gave discrepant results. The high GTLS concentration reduced the number of viable *M. bovis* below the level of detection in all but one pool when 10^3^ CFU/mL was spiked. In contrast, using the low concentration EU GTLS protocol, four out of six pools were positive in culture, suggesting that the GTLS concentration stated in the OIE Code is not high enough to eliminate even a low concentration of *M. bovis* in semen. 

Our results on efficacy of GTLS are in line with previous studies by Shin et al. [10] and Visser et al. [12,13], although there are marked differences in experimental setup among the studies. In our study, an AI straw production system was performed in a commercial facility using industrial scale procedures, the wild type study strain had been recently isolated from AI straws, different extenders and treatments were used, and survival of *M. bovis* was measured using a different method. In the earlier studies [10,12,13], animal protein containing extenders were used, as well as a plate counting method was used to detect viable *M. bovis*. Shin et al. [10] found that a high GTLS concentration in 20% egg yolk citrate extender showed 85% reduction of viable *M. bovis.* GTLS in other extenders was less mycoplasmacidal, thus, extender composition seemed to affect the efficacy of GTLS on *M. bovis.* However, the opinion of Shin et al. [10] was that the reduction of *M. bovis* concentration was so notable that it made the semen safe to use, and Shin’s GTLS protocol was implemented for use in the Unites States AI industry. Later, Visser et al. [12,13] studied the effect of high GTLS in egg yolk tris extender. They noticed a one to two decimal reduction in *M. bovis* numbers in some batches, and in some batches the number of viable *M. bovis* was even higher in the GTLS-treated semen as compared with non-treated semen. We did not attempt to analyze the number of colony-forming units after liquid nitrogen storage. Instead, we aimed to find any viable *M. bovis* cells by enrichment culture and using real-time PCR to detect *M. bovis* in broth cultures. Previously, we showed that the limit of detection of this method was 1.4 × 10^2^ CFU/mL of *M. bovis* PG45 in fresh, non-frozen extended bull semen [20]. Animal protein-free extender used in this study did not seem to enhance the efficacy of antibiotics as compared with earlier studies. The inclusion of further field strains isolated from AI semen in this study would have been appropriate, but these were not readily available.

Macrolide and linco/spectinomycin resistance, in recent *M. bovis* isolates from Europe, has increased as compared with isolates before 2000 [15,17]. This may have an impact on the effect of GTLS in *M. bovis* in semen as the highest dilutions tested in recent European studies [16,21] were from 64 to 256 µg/mL, and several strains had MIC values higher than the highest tested concentration. Antimicrobial susceptibility studies [15,16,17,21,22] showed that contemporary *M. bovis* strains are susceptible to fluoroquinolones, except for a few strains that had MIC_90_ over 32 µg/mL. Gloria et al. [18] reported that ofloxacin, a fluoroquinolone antibiotic, had non-significant effects on sperm quality and controlled bacteria efficiently in semen doses, although they did not study the effect on mycoplasmas. This tempted us to examine the effect of two different ofloxacin concentrations on *M. bovis* in semen. To our knowledge, this is the first publication on efficacy of a fluoroquinolone on *M. bovis* in commercial semen production. Although the ATCC strain used for spiking had an MIC value of 0.25 µg/mL for enrofloxacin and danofloxacin, the 100 µg/mL ofloxacin concentration in extender had no effect on the viability of the ATCC strain, and two out of three tested pools of the high ofloxacin concentration were also culture positive. Antimicrobial resistance, in the strains we used in this study did not explain the results, as MIC values for tylosin, lincomycin, and spectinomycin, as well as for fluoroquinolones, were well below the concentrations of antibiotics in semen extenders. The biological conditions for antibiotics to act with *M. bovis* in MIC testing are remarkably different as compared with conditions in semen production. 

Most antibiotics require ongoing cell activity or cell division to be able to destroy bacteria. Low temperature can keep bacteria in a stationary phase of growth, thus, making the antibiotics almost ineffective. This is considered in the EU directive 88/407/1993 which states that extended semen with antibiotics must be kept a minimum of 45 min at 5 °C, and in the CSS protocol that requires, first, adding antibiotics in raw semen, and then keeping extended semen at 5 °C for a minimum of two hours before freezing. In our study, extended semen with different antibiotics was kept for 3–3.5 h at temperatures (decreasing from 34 °C to 17 °C) that, in theory, allowed replication of mycoplasmas. Thus, the negative effect of low temperature on antimicrobial effect cannot explain our results.

A possible way to control the dissemination of *M. bovis* via AI could be testing of raw semen or multiple straws of extended semen using PCR. However, PCR inhibitors present in semen can pose problems for detecting *M. bovis*. Semen contains very high amounts of DNA and protein, potassium ions, citric acids, and fructose. Therefore, it is essential to have a highly sensitive method for DNA isolation from bull semen. We compared three different DNA extraction methods and found that InstaGene™ proved to be the most efficient and robust method to detect *M. bovis* DNA in extended bull semen. To our knowledge, Parker et al. [23] and McDonald [24] are the only studies on the sensitivity of real-time PCR detection of *M. bovis* in semen. Parker et al. [23] used the Triton-X extraction method which, in our study, had lower sensitivity than the InstaGene™ method. Together with the *uvrC* gene-based real-time PCR, their limit of detection was 1.3 × 10^5^ CFU/mL, which was higher than for our method. McDonald [24] used a commercial DNA isolation kit on spiked semen and multiplex real-time PCR targeting *fusA* and *oppD/F* genes. These assays detected 3.1 × 10^3^ *M. bovis* genomes per mL semen, which was a similar level of detection to our InstaGene™ method. Little is known about shedding of *M. bovis* into semen during different stages of infection. It is generally known that shedding of mycoplasmas into semen can be intermittent. Ball et al. [25] showed that at least three semen lots from a bull needed to be analyzed to find out if the bull was shedding mycoplasmas into semen. This has also been shown for the secretion of *M. bovis* to semen. A clinically healthy bull in the AI center was shown to shed *M. bovis* in semen for a very short period and intermittently [5]. Our culture and PCR results also highlight the problem that *M. bovis* seems to be unequally distributed in extended semen, a phenomenon we also saw when examining the straws from the naturally infected bull semen. Therefore, it is important to analyze several straws, even from the same lot, when trying to detect *M. bovis* in semen. We also found that, within the same lot, some straws were positive only in PCR, but unculturable [5]. This can lead to unnecessary disposing of semen lots that contain only dead bacteria.

AI using *M. bovis*-contaminated semen can introduce the agent into naïve dairy herds. We showed that even using modern commercial extender and industrial procedures, neither GTLS nor ofloxacin reached 100% bactericidal effect on *M. bovis*. Our results suggest that regarding *M. bovis* in semen, it is safer to use the high 500/100/300/600 µg/mL GTLS concentration. To be able to fully understand the risk of *M. bovis* contaminated semen in dairy herds and to know if it is even necessary to have zero tolerance for *M. bovis* in commercial semen, we need to know what is the *M. bovis* load that would initiate a pathological process in the female genital system.

Another option, although very laborious, is to test processed semen for the presence of *M. bovis*, considering the special features of *M. bovis* infection in bulls and occurrence in semen. However, the increasing antimicrobial resistance in contemporary *M. bovis* strains, the difficulties achieving 100% mycoplasmacidal effect using antibiotics in semen, and the pressure to reduce the amount of antibiotics used in semen industry calls for future attempts to allow only *M. bovis* negative bulls into semen production. 

## 4. Materials and Methods

### 4.1. Semen Collection and Quality Assessment

All studies were done in a commercial AI straw producing AI center’s laboratory using industrial scale procedures. This was possible because the semen production ceased in this center after these experiments. Semen from three bulls was collected into sterile collection tubes at the AI center of VikingGenetics, Hollola, Finland. The motility of each semen batch was evaluated microscopically at 200× magnification using prewarmed glass slides and coverslips. Viability and concentration of each batch was analyzed using flow cytometry (CyFlow, Partec, Germany). Pooled raw semen (0.3 mL) was cultured in Friis broth [26] to detect possible *Mycoplasma* contamination. The final sperm cell concentration was 12–13 million per straw. On the basis of the weight and concentration, the volume of extender was calculated.

### 4.2. Mycoplasma Bovis Strains 

Two *M. bovis* strains were used in spiking, i.e., a wild type isolate from commercial AI straws (strain 198, [5]) and a reference strain ATCC 27368. Strains were cultured in Friis broth in closed tubes at 37 °C, for 70 ± 2 h. High (10^8^ CFU/mL) and low (10^5^ CFU/mL) concentration stock solutions were made from the cultures in Friis broth. To verify the *M. bovis* concentration of the stocks, ten-fold dilutions were made and plated on Friis plates. Plates were incubated at 37 °C, in 5% CO_2_, for 7 days, and colony-forming units were counted. 

### 4.3. Protocols Used for Processing Semen

Semen from the three bulls was pooled and divided into 30 aliquots which were kept at 32 °C. Commercial animal protein-free extender base containing 7% glycerol was used in all protocols. Six antibiotic protocols were compared as follows: (1) GTLS (500/100/300/600 μg/mL, respectively) fresh antibiotic supplemented extender; (2) raw semen was treated with GTLS fresh antibiotics for 3 min and further extended with GTLS (500/100/300/600 μg/mL, respectively) fresh antibiotic supplemented extender (according to Certified Semen Services (CSS) requirements), later called CSS GTLS; (3) GTLS (250/50/150/300 μg/mL, respectively), antibiotic supplemented extender (ready to use liquid concentrate containing antibiotics), according to the OIE Code, Article 4.7.7, later called EU GTLS; (4) ofloxacin 100 μg/mL (Sigma Aldrich 33703) antibiotic supplemented extender; or (5) ofloxacin 400 μg/mL antibiotic supplemented extender; and (6) extender without antibiotics, control. The final concentration of the *M. bovis* strains in extended semen was either 10^6^ CFU/mL or 10^3^ CFU/mL. Friis broth was used as a negative control in each different antibiotic/extender aliquot. The protocols are described in Table 5. All extenders, antibiotics, and Friis broth were kept at 32 °C before being added into the semen. All protocols, except number two (CSS GTLS), included diluting the semen in 1:1extender (with or without antibiotics) and Friis broth containing either 10^8^ or 10^5^ CFU *M. bovis* ATCC or wild type. In protocol two (CSS GTLS), GTLS was first diluted 1:4 in sterile water and 38 µL added into neat semen (380 µL), *M. bovis* culture (118 µL) yielding the same antibiotic concentration as if 20 µL GTLS mixture (500/100/300/600) would have been added directly to raw semen. After 3 min of incubation at 32 °C, the semen was further diluted 1:1 with extender containing GTLS. Then, all aliquots were incubated for one hour at 34 °C, after which they were diluted further with extender with or without antibiotics to give the final concentration of 56 million sperm cells/mL. Then, the temperature of the aliquots was allowed to stabilize to room temperature (approximately one hour) after which automatic semen straw filling and sealing machine (MPP Quattro, Minitube, Germany) was used. Semen was packed into 0.25 mL straws. After packing, the straws were cooled to 17 °C for one hour and further cooled quickly to 4 °C. The straws were kept at 4 °C overnight and they were deep-frozen with industrial semen straw freezer (Digitcool 5300, IMV, France) the next morning. Cryopreserved straws were stored in liquid nitrogen storage tank (−196 °C) until they were analyzed.

### 4.4. Semen Quality Parameters after Thawing

After 18 days storage in liquid nitrogen, two straws from each trial lot were thawed. The motility was assessed under phase contrast microscope. Flow cytometric analysis was used to evaluate viability and concentration of sperm cells.

### 4.5. Viability Testing of M. Bovis from Semen Straws Stored in Liquid Nitrogen

After storage of five weeks in liquid nitrogen, 18 straws from each of the 30 trial lots were randomly retrieved from the nitrogen tank. They were divided into three pools, each consisting of six straws. Straws were thawed and the content of the six straws was pooled. From each pool, 0.6 mL of semen was used in three different DNA extraction procedures described in Section 4.6., and 0.3 mL of semen was placed into 2.7 mL of Friis broth. Ten-fold dilutions, up to 10^−5^, were made into Friis broth in tightly closed tubes. Broth cultures were incubated at 37 °C for 14 days. The growth and color change of the medium were monitored every other day, and broths suspected of mycoplasma growth were plated on Friis agar and tested for *M. bovis* using real-time PCR targeting *M. bovis oppD* gene [27]. From each trial lot, all broth culture dilutions from 10^−2^ to 10^−4^ were tested for *M. bovis*, as described above, at the latest, immediately after the 14-day incubation period.

### 4.6. DNA Extraction from Semen Straws after Storage in Liquid Nitrogen

Three different protocols to extract DNA from spiked semen were compared. In each method, 200 µL of semen was used as a starting material. Method one was automated DNA extraction using a QIAcube (Qiagen, Hilden, Germany) robot and blood and body fluids protocol with QIAamp DNA mini kit. The elution volume was 150 µL. In method two [25], 200 µL of semen was combined with 200 µL of 2% Triton-X 100 (Sigma Aldrich) in 10 mM Tris and 1 mM EDTA (pH 8) buffer. The sample was thoroughly vortexed and pelleted at 13,000× *g* for 5 min. DNA was extracted from the pellet using the QIAcube robot and bacterial pellet protocol with QIAamp DNA mini kit. The elution volume was 150 µL. Method three was modified from the OIE Terrestrial Manual method to isolate DNA from bovine semen for herpesvirus PCR (chapter 3.4.11, adopted May 2017). In method three, 200 µL of semen was centrifuged at 13,000× *g* for 10 min and supernatant was discarded. The pellet was mixed with 200 µL of InstaGene™ matrix (Bio-Rad, Helsinki, Finland), 5.8 µL proteinase K (20 mg/mL), and 7.5 µL DL-dithiothreitol (1 M). Samples were incubated at 56 °C, for 30 min, and then vortexed at high speed for 10 s. The tubes were boiled in water bath (100 °C) for 8 min, and then vortexed at high speed for 10 s. Then, the tubes were centrifuged at 10,000× *g* for 3 min. The supernatant was transferred into a new microtube and stored at −20 °C. 

### 4.7. M. bovis Real-Time PCR

Broth cultures and DNA extracted from semen straw pools were examined by real-time PCR (CFX96 Touch Real-Time PCR Detection System, Bio-Rad, CA, USA) targeting *opp*D-gene of *M. bovis*, as described previously by Sachse et al [27]. Friis broth cultures were prepared for real-time PCR as follows: First, 200 µL of broth culture was incubated for 15 min at 95 °C and centrifuged at 10,000× *g* for 5 min. Two µL of culture supernatant or DNA was used as PCR template. Commercially available plasmid pUC19 was used as internal amplification control, according to Fricker et al. [28]. 

### 4.8. Determining Minimal Inhibitory Concentration (MIC) of Wild Type Strain and ATCC 27368

MICs were determined using custom made Sensititre plates (Thermo Fisher Scientific, UK). Antibiotics tested were tylosin (concentration range 0.5–32 µg/mL), lincomycin (0.25–32 µg/mL), spectinomycin (2–128 µg/mL), enrofloxacin (0.03–2 µg/mL), and danofloxacin (0.03–2 µg/mL). Testing was done according to [15,16]. Briefly, a suspension containing 5% growth indicator alamarBlue (Thermo Fisher Scientific, United Kingdom) in Friis broth without antibiotics and *M. bovis* 5 × 10^5^ CFU/mL was made, and 200 μL of the suspension was pipetted into each well of the Sensititre plate. Plates were sealed and incubated at 37 °C for 48 ± 1 h and read visually; blue indicating no growth and red indicating growth of the isolate. MIC was the lowest concentration of antibiotic completely suppressing growth (blue color).

## Figures and Tables

**Table 1:** Semen quality parameters of raw and spiked semen.

Semen	Strain (CFU/mL)	Motility %	Viability %	Sperm Concentration (10^6^/mL)
Raw semen		75.0	82.5	1850
Processed semen	ATCC 10^3^	55 ± 2.9	53 ± 3.0	65 ± 2.9
	ATCC 10^6^	56 ± 6.1	53 ± 2.5	67 ± 2.3
	wild type 10^3^	52 ± 5.5	52 ± 2.1	67 ± 2.1
	wild type 10^6^	57 ± 5.5	52 ± 4.6	67 ± 2.3
	unspiked	53 ± 5.1	54 ± 2.3	61 ± 1.3

**Table 2:** Detection of *M. bovis* wild type and ATCC 27368 by culture (+/-) from three parallel pooled samples (e.g., + + +) from different antibiotic/extender protocols after five-week storage of the straws in liquid nitrogen. Concentration used in spiking and culture dilution are shown in the table.

	10^3^ CFU/mL			10^6^ CFU/mL		
Culture dilution	−2	−3	−4	−2	−3	−4
Wild type strain						
GTLS 500/100/300/600 ^a^	- - -	- - -	- - -	- - -	+ + +	+ + +
CSS GTLS ^b^	- - -	- - -	- - -	- - -	- - -	+ + +
EU GTLS ^c^	- - -	- - +	- - -	- - -	+ + +	+ + +
OF 400 µg ^d^	- - -	- - -	- - -	- - -	+ + +	+ + +
OF 100 µg	- - -	- - -	- - -	- - -	+ + +	+ + +
no antibiotic	+ + +	+ - +	+ - -	+ + +	+ + +	+ + +
ATCC strain						
GTLS 500/100/300/600	- - -	- - -	- - -	+ - -	- - -	+ + +
CSS GTLS	- - -	- - -	- + -	+ + +	- + -	+ + +
EU GTLS	- - -	+ + +	+ - -	- - -	+ + +	+ + +
OF 400 µg	- - -	- - +	+ - +	+ - +	+ + +	+ + +
OF 100 µg	- - -	+ + +	- + +	+ - -	+ + +	+ + +
no antibiotic	+ + +	+ + +	+ + -	+ + +	+ + +	+ + +

^a^ gentamycin (500 µg/mL), tylosin (100 µg/mL), lincomycin (300 µg/mL), spectinomycin (600 µg/mL); ^b^ Certified Semen Services gentamycin (500 µg/mL), tylosin (100 µg/mL), lincomycin (300 µg/mL), spectinomycin (600 µg/mL) protocol; ^c^ gentamycin (250 µg/mL), tylosin (50 µg/mL), lincomycin (150 µg/mL), spectinomycin (300 µg/mL); ^d^ ofloxacin.

**Table 3:** Comparison of three different DNA extraction methods for detection of *M. bovis* in semen using *oppD* real-time PCR. Results (+/-) from three parallel pools (e.g., + + +) from each antibiotic protocol are shown.

	10^3^ CFU/mL						10^6^ CFU/mL					
	ATCC			Wild Type			ATCC			Wild Type		
	Method 1 ^a^	Method 2 ^b^	Method 3 ^c^	Method 1	Method 2	Method 3	Method 1	Method 2	Method 3	Method 1	Method 2	Method 3
GTLS 500/100/300/600	- + +	+ + -	+ + +	+ - -	+ - +	+ - -	+ + +	+ + +	+ + +	+ + +	+ + +	+ + +
CSS GTLS	+ - -	- + +	+ + +	- + +	- - -	+ + +	+ + +	+ + +	+ + +	+ + +	+ + +	+ + +
EU GTLS	+ + +	+ + -	+ + +	+ + +	+ - +	- + +	+ + +	+ + +	+ + +	+ + +	+ + +	+ + +
OF 400 µg	+ + +	+ - +	+ + +	+ - +	- - -	+ + -	+ + +	+ + +	+ + +	+ + +	+ + +	+ + +
OF 100 µg	- + -	- - +	+ - +	- + -	- - -	+ + +	+ + +	+ + +	+ + +	+ + +	+ + +	+ + +
Control	- + +	- - +	+ + +	- + +	+ + -	+ - +	+ + +	+ + +	+ + +	+ + +	+ + +	+ + +

^a^ QIAamp DNA mini kit (Qiagen, Hilden, Germany); ^b^ 2% Triton-X 100 and QIAamp DNA mini kit (Parker et al. 2017); ^c^ InstaGene™ matrix (Bio-Rad, Helsinki, Finland).

**Table 4:** MIC values (µg/mL) of ATCC 27368 and wild type strains (dilution range of antibiotic tested).

*Antibiotic*	Dilution Range Tested µg/mL	ATCC 27368	Wild Type
Tylosin	0.5–32	≤0.5	16
Lincomycin	0.25–32	2	1
Spectinomycin	2–128	4	≤2
Enrofloxacin	0.03–2	0.25	0.25
Danofloxacin	0.03–2	0.25	0.25

**Table 5:** Antibiotic/extender protocols used for processing semen.

GTLS (500/100/300/600)	CSS GTLS (500/100/300/600)	EU GTLS (250/50/150/300)	OF400	OF100	Control
0.38 mL semen	0.38 mL semen	0.38 mL semen	0.38 mL semen	0.38 mL semen	0.38 mL semen
+	+	+	+	+	+
0.38 mL GTLS extender	0.118 mL *M.bovis*	0.38 mL GTLS extender	0.38 mL OF extender	0.38 mL OF extender	0.38 mL extender
					no antibiotic
+	+	+	+	+	+
0.118 mL *M. bovis*	38 µL GTLS 1:4	0.118 mL *M. bovis*	0.118 mL *M. bovis*	0.118 mL *M. bovis*	0.118 mL *M. bovis*
	3 min				
+	+	+	+	+	+
	0.38 mL GTLS extender +				
1 h 34 °C +	1 h 34 °C +	1 h 34 °C +	1 h 34 °C +	1 h 34 °C +	1 h 34 °C +
10.875 mL GTLS extender	10.875 mL GTLS extender	10.875 mL GTLS extender	10.875 mL OF extender	10.875 mL OF extender	10.875 mL extender
					no antibiotic
Room temperature 50–90 min and packing in straws (0.25 mL per straw)					
1 h + 17 °C					
21 h + 4 °C					
Storage in liquid nitrogen					
